# Preparation and Characterization of Copper-Crosslinked Alginate–Hyaluronic Acid Aerogels as Potential Wound Dressing Materials with Enhanced Antibacterial Properties

**DOI:** 10.3390/polym17172406

**Published:** 2025-09-04

**Authors:** Tamara Athamneh, Mohammad A. A. Al-Najjar, Raghad Garafat, Alaa Mahmood Abuawad, Areen Alshweiat, Muna Barakat, Wael Fatehi Abu-Irmaileh, Adel Maher Hamdan, Tasneem Ali Odat, Razan Altarabeen, Yamen Bani Younes, Irina Smirnova

**Affiliations:** 1 Institute of Nanotechnology, Jordan University of Science and Technology, Irbid 22110, Jordan; rygrafat20@nano.just.edu.jo (R.G.); taodat20@nano.just.edu.jo (T.A.O.); 2 Department of Pharmaceutical Sciences and Pharmaceutics, Faculty of Pharmacy, Applied Science Private University (ASU), Amman 11937, Jordan; moh_alnajjar@asu.edu.jo (M.A.A.A.-N.); a_abuawad@asu.edu.jo (A.M.A.); 3 Department of Pharmaceutics and Pharmaceutical Technology, Faculty of Pharmaceutical Sciences, The Hashemite University, Zarqa 13133, Jordan; areen.alshweiat@hu.edu.jo; 4 Department of Clinical Pharmacy and Therapeutics, Faculty of Pharmacy, Applied Science Private University (ASU), Amman 11937, Jordan; m_barakat@asu.edu.jo; 5 Quatro-P4 Company for Industrial and Commercial Investment, Amman 11947, Jordan; w.irmaileh@quatro-p4.com (W.F.A.-I.); a.hamdan@quatro-p4.com (A.M.H.); 6 Institute of Thermal Separation Processes, Hamburg University of Technology, Eissendorfer Strasse 38, 21073 Hamburg, Germany; irina.smirnova@tuhh.de; 7 Department of Psychology, University of California Los Angeles, Los Angeles, CA 90095-1563, USA; yamenby@g.ucla.edu

**Keywords:** antibacterial aerogel, wound healing, copper, hyaluronic acid–alginate composite

## Abstract

The development of advanced wound dressing materials with enhanced antibacterial properties is critical for improving patient outcomes and reducing infection risks. This study introduces a novel bio-based aerogel composed of copper-crosslinked alginate and hyaluronic acid, synthesized using supercritical gel drying techniques. Alginate and hyaluronic acid polymers are widely used in the pharmaceutical and medical industries because of their nontoxicity, biodegradability, and biocompatibility. This study aimed to create an aerogel that could be used as a potential wound dressing material by crosslinking hyaluronic acid and alginate with copper. The bio-based aerogel was prepared by ionic gelation and supercritical gel drying. The prepared materials were characterized using scanning electron microscopy (SEM), Fourier transform infrared spectroscopy (FTIR), BET surface area analysis, and energy-dispersive X-ray fluorescence (XRF). Moreover, the aerogel wound dressing properties were evaluated in terms of fluid uptake and antibacterial activity against *S. aureus* and *E. coli*. The physicochemical characterization of the prepared aerogels revealed their unique structural and morphological features, which are influenced by copper ion concentration and crosslinking time. Regarding their wound dressing evaluation, both aerogel and hydrogel were found to have antibacterial properties when tested on *S. aureus* with inhibition zones of (36 mm, 23 mm) and *E. coli* (31.6 mm, 21 mm) for hydrogel and aerogel, respectively. Also, excellent fluid uptake was found to reach up to 743%. These findings underscore the potential of copper-crosslinked alginate–hyaluronic acid aerogels as innovative wound dressing materials that combine superior antibacterial efficacy with excellent fluid management, paving the way for improved wound healing solutions.

## 1. Introduction

The skin is the largest organ in the human body. It typically covers an area of around 2 m^2^ of the human body [1,2]. The skin’s primary function is acting as a barrier, insulating inside organs against microorganisms and UV radiation while regulating body temperature. Skin additionally promotes the body’s immune system and sensory detecting processes [3,4]. A wound is a rupture in the skin’s epithelial integrity that can cause alteration of the structure and function of surrounding normal tissue [5]. The wound is categorized as acute or chronic based on the healing process and duration time [6]. Acute wounds happen due to unanticipated accidents or surgical wounds and heal within an expected time frame of 8 to 12 weeks. The degree of damage in the various skin layers affects how long it takes for the wound to heal.

On the other hand, chronic wounds, which are typically caused by burns, decubitus ulcers, and leg ulcers, fail to heal in a predictable time frame [4,7]. Wound healing is generally split into four phases: hemostasis, inflammation, proliferation, and tissue remodeling [8]. The majority of chronic wounds are in a chronic inflammatory state. According to previous studies, the incidence of microbial colonization and proliferation in chronic wounds triggers a continuous influx of polymorphonuclear leucocytes, which results in the release of cytotoxic enzymes, oxygen-free radicals, inflammatory mediators, and matrix metalloproteases, which cause extensive localized tissue damage in the patient [9,10].

Conventional wound dressings, like bandages and gauze, are widely accessible, inexpensive, and easy to use in medical practices to manage bleeding. It allows wound exudate to be absorbed while functioning as a protective barrier [11]. On the other hand, conventional wound dressings have weak hemostatic properties. They are quickly contaminated by tissue fluids and may adhere to wounds upon prolonged contact with the wound. The aforementioned drawbacks highlighted the need for innovative wound dressings such as hydrogels, aerogels, films, and nanofibers [11,12]. Nevertheless, dressings with intrinsic antibacterial qualities are more desirable due to their long-lasting antimicrobial effects [13,14].

Aerogels, as a synthetic solid material, offer exceptional and frequently unprecedented physical qualities, such as ultralow density (0.0001 to 0.2 g/cm^3^), excellent porosity, and high surface areas < 200 m^2^ g [15,16,17]. Because of their unique features, aerogels have a wide range of applications such as wound dressing, energy storage, catalysis, piezoelectric, thermoreceptors, and sensors [18,19,20]. As the lightest manufactured solid compounds on the earth with the highest empty volume percentage, aerogels are strong candidates for evaluating their potential as drug delivery systems [15]. Aerogel’s porous structure offers many advantages to the wound-healing matrix [21,22]. The porous design of aerogel allows for the holding of absorbed liquids without re-leaking [23]. Moreover, aerogels have additional characteristics, such as excellent breathability, minimal adhesion, minimizing the harm to newly produced tissue during the healing process, and exceptional thermal insulating capabilities, particularly in burn wounds; this can assist in maintaining the temperature in the wound site and protect it from excessive fluctuations in temperature [22,23].

Among the different types of aerogels, biopolymer aerogels stand out as a promising choice for wound dressing due to their low toxicity, high degree of biological compatibility, and excellent biological activity [24]. Moreover, research on biopolymers and biopolymer-composite aerogels has exceedingly increased in the past ten years because of the need for more sustainable precursors, special and adjustable features, and ease of functionalization [21]. Several biopolymers such as alginate, hyaluronic acid, chitosan, cellulose, agar, and collagenDI are commonly used in wound dressing [20,22,24,25,26].

HA, also known as hyaluronan, is a compound made up of repeated disaccharide molecules of β-d-glucuronic acid and N-acetyl-d-glucosamine that are joined together by β-1,3 and β-1,4 glycosidic linkages [27]. The HA structure’s abundance of carboxyl and hydroxyl groups is responsible for its very hydrophilic nature. This property enables HA to absorb exudates and improve cell adherence. Furthermore, HA’s biocompatibility, biodegradability (through the enzymatic activity of hyaluronidases), and ease of chemical modification promoted its use in wound healing to promote hemostasis, regulate inflammation, and encourage re-epithelialization [28,29,30].

Alginate (ALG) is a common polysaccharide made up of two isomers, β-D-mannuronic acid (M) and α-L-guluronic acid (G) [31]. These two residues are connected by 1,4 glycosidic linkages, forming three distinct block structures in ALG: poly M blocks, poly G blocks, and poly MG blocks [31,32]. The physicochemical features of ALG are greatly reliant on its structure, such as the molecular weight, M/G ratio, arrangement of M and G residues, and degree of acetylation it has a substantial impact on gel formation, viscosity, and interaction with other molecules. Additionally, the structural characteristics are affected by their sources and growing circumstances [33].

Topical antimicrobials can be used with wound dressings to prevent or treat infections. Preliminary research indicates that ALGs may crosslink with polyvalent cations such as aluminum, copper, and zinc by egg-box model, which may exhibit antibacterial properties [34]. The “egg-box” concept is well recognized as the general gelation process of ALG [35]. Copper is a vital element in the body that has a substantial impact on many physiological functions [36]. It is a powerful antioxidant capable of removing free radicals from tissues and protecting cells, and it additionally has a role in enhancing wound healing [37].

The gelation of ALG by Cu^2+^ occurs in a manner that differs significantly from Ca^2+^ gelation. During this process, four oxygen atoms from two negatively charged carboxyl groups and two neutrally charged carboxyl groups, along with one central Cu^2+^ ion, combine to form the egg-box structure of Cu-ALG. The egg-box dimer is always created when Cu^2+^ and ALG are combined, resulting in a consistent crosslinking pattern that is longer than one formed with Ca^2+^. In contrast, Ca-ALG dimers are short and result in a random crosslinking of Ca and ALG [38]. The affinity of Cu^2+^ to ALG chains is approximately ten times stronger than that of Ca^2+^, as demonstrated by Haug and Smidsrd [39]. When ALG and Cu^2+^ come into contact, they quickly form a thick gel layer that prevents further dispersion of Cu^2+^ through the gel [38]. Copper was chosen in this study as the crosslinking agent because it creates a stronger and more reliable bond with ALG, resulting in a stable gel that forms quickly and controls the release of copper ions effectively. Beyond its structural advantages, copper also offers important health benefits—it acts as a powerful antioxidant and supports faster wound healing, making it a better choice than other similar ions that might not provide these added effects.

Copper-based wound dressings have shown promising advantages over other traditional dressings. This may be referred to its potent broad-spectrum antimicrobial properties and its physiological role in wound healing by promoting angiogenesis, granulation tissue formation, and skin regeneration [40] In contrast, conventional wound dressings that contain silver lack a biological role in tissue repair and have been associated with cytotoxic effects on keratinocytes and fibroblasts, which can delay the wound healing process [41]. In a recent clinical study of Gorel et al., it was reported that the application of copper dressings in situ for noninfected wounds results in the stimulation of the wound healing processes, as opposed to silver dressings [42].

The key contribution of this work is the development of a novel smart wound dressing material based on copper-crosslinked ALG/HA aerogels, which possess both fluid uptake capabilities and antibacterial activity. This new combination provides multiple benefits, where the need to apply antibacterial is no longer required. Also, the aerogel transforms into a gel material after absorbing the wound exudates, which offers painless dressing removal. Moreover, the presence of hyaluronic acid is expected to enhance the wound healing process.

The goal of this research is to study copper-crosslinked ALG/HA aerogels as innovative wound dressing materials, studying copper as a crosslinker and an antibacterial agent to create an innovative wound dressing material with antibacterial activity and excellent fluid uptake.

## 2. Materials and Methods

### 2.1. Materials

Sodium ALG (ALG) (catalog no. 71238) was supplied by Sigma Aldrich (Munich, Germany). Hyaluronic acid (HA), sodium salt, cosmetic grade, (Mw  =  9.9 × 10^5^ Da) was purchased from Xi’an Trend Biotechnology (Xi’an, China). Copper sulfate was supplied by the Manaseer Group (Amman, Jordan). Ethanol of 99.8% purity was provided by Solvochem-Holland (Rotterdam, The Netherlands); carbon dioxide (CO_2_) with a purity of 99.99% was provided by the Jordanian Gas Co. (Amman, Jordan); Mueller–Hinton agar (lot no. EMHT110422013) was purchased from Biolab for splendid isolation (Budapest, Hungary); Mueller–Hinton broth was supplied by Oxoid (Basingstoke, UK); and Dulbecco’s phosphate-buffered saline (PBS) (lot no. CP16-1019) was supplied by Capricorn scientific (Ebsdorfergrund, Germany).

### 2.2. Preparation of Stock Solution of ALG and Stock Solution of HA

To produce stock solutions, sodium alginate (ALG) was suspended in deionized water at a concentration of 2% (*w*/*w*) and magnetically stirred at room temperature until a homogeneous solution was achieved. Meanwhile, 2% (*w*/*w*) HA was dissolved in deionized water and also magnetically stirred.

### 2.3. Alginate/Hyaluronic Acid Hydrogel Preparation

To prepare the ALG/HA hydrogel, the ALG and HA solutions were mixed (1:1 volume ratio) at 1200 rpm until completely blended. The mixture (ALG-HA) was submerged in copper solutions (1%, 2%, 3%, 4%) at different times of gelation (4 h, 8 h, 24 h, 48 h). After gelation, the hydrogels were cut into disks of a diameter of 18 and a thickness of 10 mm using a sharp medical blade.

### 2.4. Alcogel and Aerogel Preparation

**Figure 1:**
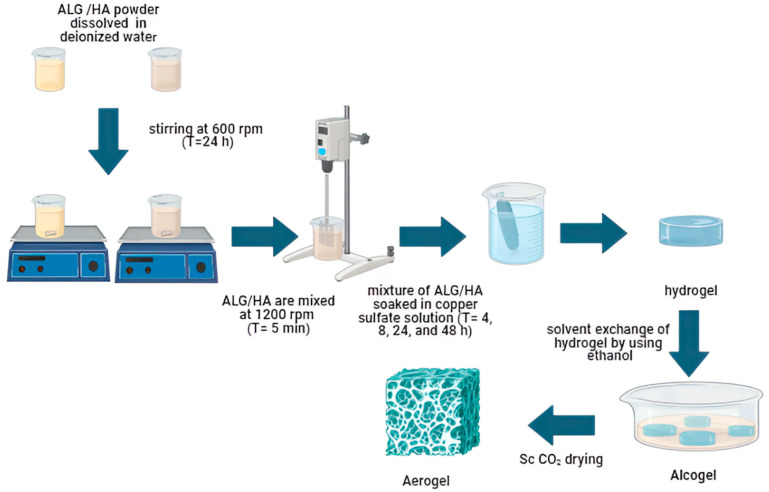
Scheme illustrating the main steps of preparation of ALG/HA with Cu^2+^.

For the solvent exchange step, hydrogel samples were submerged in ethanol–water mixture increasing from 70, 90, to 100% *v*/*v*, and each step of solvent exchange lasted for 24 h [40].The alcogel samples were dried using supercritical CO_2_ at a temperature of 50 °C and a pressure of 120 bar. A continuous flow of CO_2_ (20–80 g/min) was maintained until the ethanol was fully extracted, which took approximately 5 h. Finally, slow depressurization of the autoclave (1–3 bar/min) was performed. The autoclave was evacuated once ambient pressure was reached, and the samples were collected. The main steps in the preparation of the aerogel are summarized in Figure 1.

## 3. Physical and Chemical Characterization of Hydrogel and Aerogel

### 3.1. Fourier Transform Infrared Spectroscopy (FTIR)

The identification of functional groups of ALG, HA, copper, hydrogel, and aerogel was obtained by Vertex 80v FTIR spectrometer (Bruker company, Billerica, MA, USA) with a range from 4000 to 400 cm^−1^ to identify the chemical (functional) groups.

### 3.2. Scanning Electron Microscopy (SEM)

The morphology and the porosity of ALG-HA aerogels were analyzed by Scanning Electron Microscopy (SEM) Quanta FEG SEM 450 (ThermoFisherScientific, Brno, Czech Republic), operated under a high vacuum with an accelerating voltage of 10 KV. Before analysis, the samples were cut open and coated with a conductive, thin gold layer (approximately 5 nm) using a Sputter Coater (Quorum Q 150R).

### 3.3. Nitrogen Physisorption (BET Method)

The specific surface area of aerogel was evaluated by the Brunauer–Emmett–Teller (BET) method. Nitrogen multilayer adsorption isotherms were studied using a Quantachrom Corporation 360 Engineering instrument (Golden, CO, USA).

### 3.4. Energy Dispersive X-Ray Fluorescence (EDXRF)

Energy Dispersive X-ray fluorescence (EDXRF) (Rigaku MODEL: Rigaku Nexqc+ QuantEZ, Tokyo, Japan) was used to measure the concentration of copper in the hydrogel at three different points: the outer, middle, and inner part of the disks. The effect of gelation time and copper sulfate concentration on the proportion of copper in the hydrogel was also studied.

### 3.5. Evaluation of the Dressing Properties

#### 3.5.1. Fluid Uptake Studies

To assess the fluid uptake, swelling studies of 13 mm diameter of ALG/HA aerogels disk were conducted in 100 mL of phosphate-buffered saline (PBS) solution (pH = 7.4). At ambient temperature, the aerogel pieces were removed from the PBS after 0.5, 1, 2, 3, 4, 5, 6, 24, 48 h and weighted (excess of buffer on the surface was dabbed away with filter paper). This mass was compared with the original dry mass to determine the amount of absorbed fluid. Fluid uptake for the aerogel was expressed by using the following equation:

$$\mathrm{fluid}\;\mathrm{uptake}\%=\left(\frac{W_{s}-W_{d}}{W_{d}}\right)\times 100\%$$

where W_d_ and W_s_ are the masses of swollen and dry aerogel.

Each sample was performed three times (*n* = 3).

The fluid uptake study was conducted for the best formula, not for all samples.

#### 3.5.2. Evaluation of the Antibacterial Activity

Bacteria were cultured in nutrient broth and incubated at 37 °C, 24 h, and then the bacterial turbidity was measured using a spectrophotometer at 600 nm. The bacteria strains were controlled to a 0.5 McFarland value of normal turbidity (1.5 × 10^8^ CFU/mL). The disk diffusion Susceptibility test was used to assess the antibacterial activity of the copper ions against specified Gram-positive (*S. aureus*) and Gram-negative bacteria (*E. coli*). The bacteria were spread out on Mueller–Hinton agar, and the hydrogel and aerogel disks were then placed on agar plates with tested pathogenic strains for 24 h at 37 °C. Following a 24 h incubation period, every specimen had an obvious inhibition zone whenever there was antibacterial activity. Based on the average diameter of the clear area measured of each copper concentration loaded in hydrogel and aerogel and each time of gelation composites, the zone of inhibition of aerogel and hydrogel after 24 h of incubation at 37 °C was calculated, and the aerogel diameter was 13 mm, whereas the hydrogel diameter was 18 mm. Each test was performed in triplicate.

## 4. Results and Discussion

**Figure 2:**
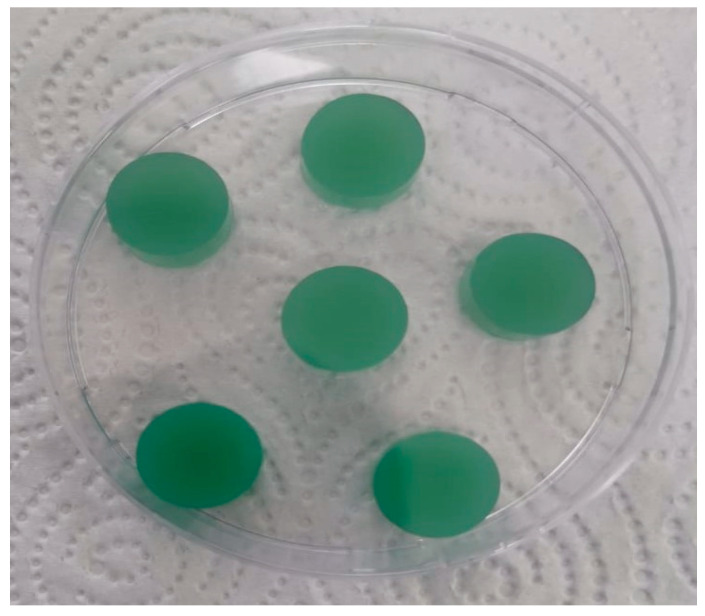
Sample of ALG/HA hydrogel (4% copper sulfate, 48 h of gelation time).

The prepared hydrogels were cut into disks (see Figure 2). Divalent cations (Cu^2+^) were used in the gelation of ALG. The “egg-box” mechanism, which describes the gelation process, involves the formation of ionic bonds between the Cu^2+^ and the G-residues of the ALG polymer chains [38]. An interpenetrating polymeric network structure was obtained to create the ALG-HA hydrogel. Cu^2+^ ions ionically crosslink the ALG network to provide structural reinforcement, and the HA network is integrated into this structure at the molecular level without forming covalent bonds [43].

### 4.1. Fourier Transform Infrared (FTIR) Spectroscopy

**Figure 3:**
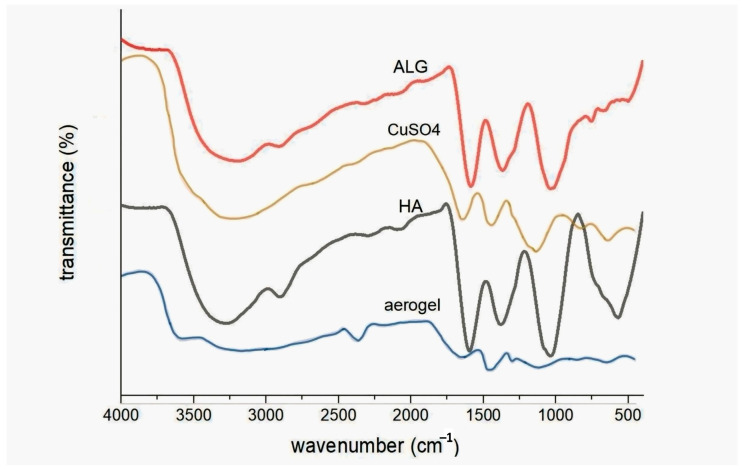
FTIR spectra of plain ALG, HA, aerogel (2%) and copper sulfate powder.

Figure 3 shows the FTIR spectra of aerogel, copper sulfate, ALG powder, and HA powder. A broad peak around 3252 cm^−1^ in the FTIR spectrum of plain ALG powder refers to the O-H stretching vibration. Meanwhile, the peak near 1598 cm^−1^ is related to the C = O asymmetric stretching of the carboxyl group, and the peak at 1407 cm^−1^ is associated with the symmetric COO stretching vibration. The peak appears at around 1609 cm^−1^ in the FTIR spectrum of plain HA, according to the C=O asymmetric stretching. In contrast, a hydroxyl group O-H broad peak appears at around 3284 cm^−1^, which corresponds to carboxyl group asymmetric stretching. The symmetric stretching of the carboxyl group COO^−^ provides the reason for the 1403 cm^−1^ peak. The aerogel caused the amide C=O asymmetric stretching shoulder at 1566 cm^−1^ to disappear, possibly due to hydrogen bond formation between the two polymers or HA dilution. Also, a peak at 1100 cm^−1^ is associated with S-O stretching vibrations, another peak at 3300 cm^−1^ is linked with O-H stretching vibrations, and a peak at 650 cm^−1^ is associated with CuO stretching vibrations.

### 4.2. Internal Morphology by SEM

**Figure 4:**
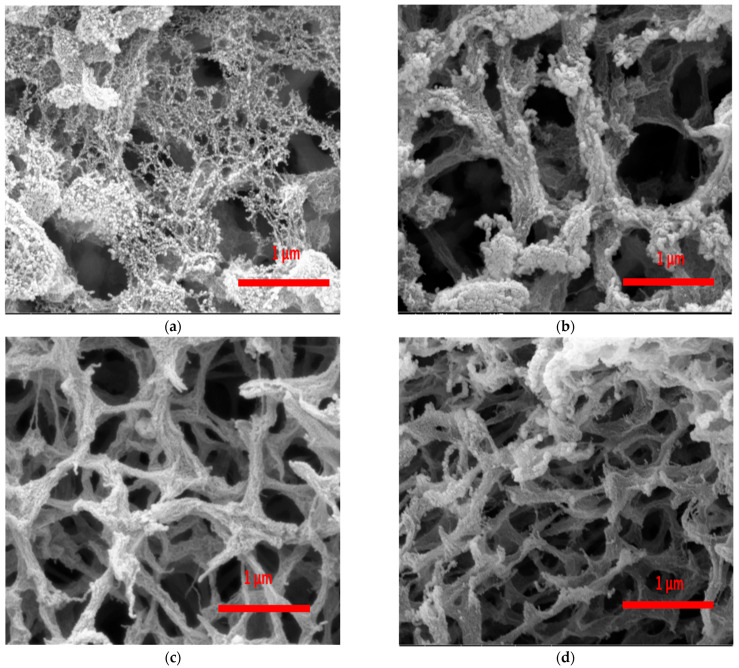
Scanning electron microscopic images of the internal section of 3% w/w Cu-ALG/HA aerogel: (**a**) 4 h, (**b**) 8 h, (**c**) 24 h, (**d**) 48 h gelation time.

The inner morphology of the 3% copper sulfate aerogel produced with varying gelation times revealed the formation of mesoporous structures with different pores, as evident in SEM images (Figure 4). As explained earlier, copper acts as a crosslinking agent during gelation, promoting interactions with ALG and leading to mesoporous structure formation. This was successfully achieved with gelation times as short as 4 h (Figure 4a). However, increasing the gelation time up to 24 h resulted in a finer microstructure (Figure 4c). The 8 h sample (Figure 4b) exhibited an intermediate morphology between 4 h and 24 h, whereas the 48 h sample (Figure 4d) showed a denser and more compact network with smaller pores. Longer gelation times were found to negatively impact the microstructure, with 24 h standing as the optimum. This allowed copper to diffuse into the ALG/HA solution, crosslinking and forming a microstructure with pore sizes of approximately 500 nm.

### 4.3. Surface Area Analysis by BET Method

The BET surface area analysis is illustrated in Table 1.

The 2% ALG/HA aerogels that underwent 4 h gelation time showed a BET-specific surface area ranging between 325 ± 29 and 246 ± 24 m^2^/g. The 2% ALG/HA aerogels that spent 24 h gelation time showed a BET-specific surface area ranging between 211 ± 34 and 173 ± 16 m^2^/g. It was noticed that a prolonged gelation time and increased copper concentration in the gelation bath led to a decrease in the surface area. This may be attributed to structural changes during the gelation process, as longer gelation times lead to increased crosslinking density and shrinkage of the wet gel matrix, which reduces the porosity and accessible surface area of the resulting aerogel, and this was also obvious in the SEM images (see Figure 4). The result also revealed that BET surface area of the produced copper-crosslinked ALG-HA is lower than the calcium-crosslinked ALG-HA that is reported in the literature [44]. The literature indicates that ALG can be crosslinked with bivalent cations like Cu, Ca, Ba, etc., with chemical affinity between ALG and these ions increasing in the order Pb > Cu > Cd > Ba > Sr > Ca > Co. Consequently, the affinity of Ca for ALG is lower than that of Cu. This results in a faster reaction when CuSO_4_ is used as the crosslinking agent, leading to the formation of a more compact structure that is characterized by a lower specific surface area [45].

### 4.4. Energy Dispersive X-Ray Fluorescence (EDXRF)

**Figure 5:**
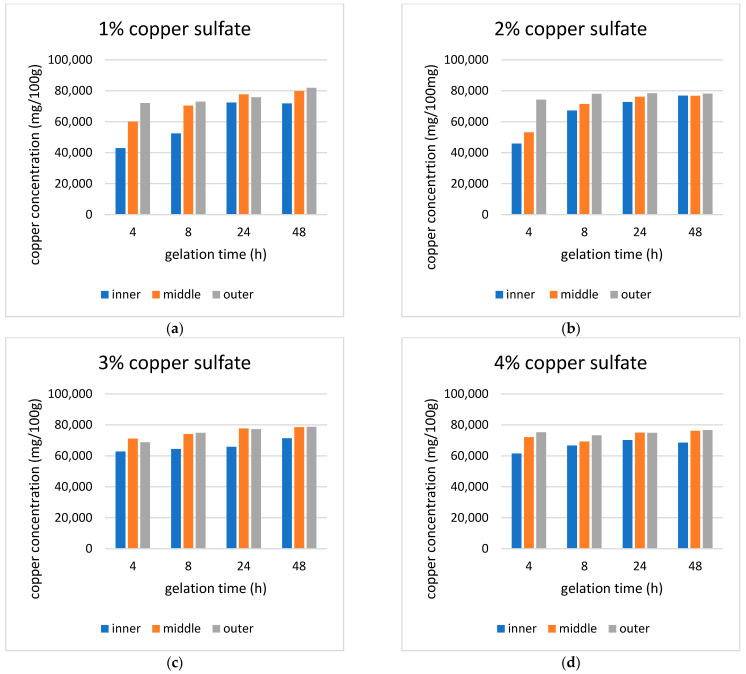
(**a**–**d**) EDXRF result of 1%, 2%, 3%,4% Cu-ALG/HA hydrogel with different gelation times, respectively.

During gelation, copper particles are transferred from the copper sulfate solution through the dialysis membrane to the inner ALG/HA solution. This was accomplished through the diffusion method, and the driving force was a concentration gradient. Diffusion occurs spontaneously, with copper particles naturally moving from areas of higher concentration to areas of lower concentration. The homogeneity of copper distribution was further studied using Energy Dispersive X-ray Fluorescence (EDXRF) (Figure 5) to track the copper concentration over time in three sections of the hydrogel: outer, middle, and inner.

### 4.5. Evaluation of the Dressing Properties

#### 4.5.1. Fluid Uptake Studies

**Figure 6:**
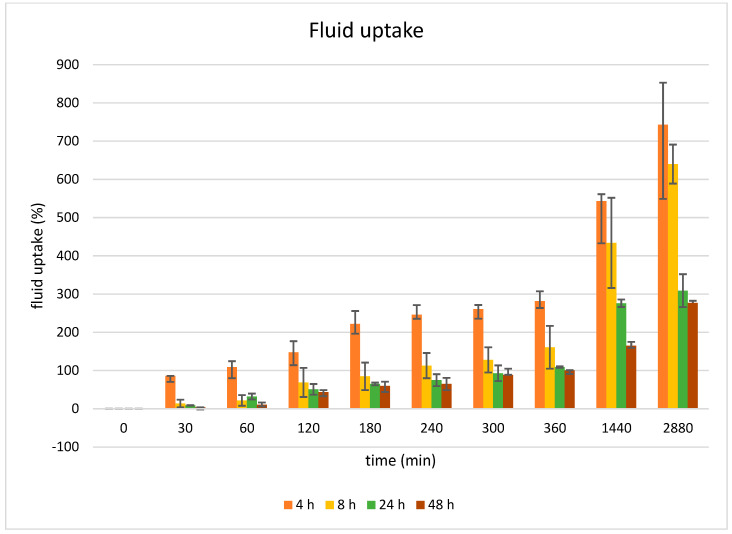
Fluid uptake of 3% Cu-ALG/HA aerogel at 4 h, 8 h, 24 h, 48 h times of gelation, means ± SD, *n* = 3.

The aerogel’s fluid uptake, which is related to its porosity and structural characteristics, refers to its capacity to absorb or retain liquids. Therefore, the influence of gelation time on the fluid uptake of 3% Cu-ALG/HA aerogels was investigated (Figure 6). Fluid uptake at gelation times of 4 h, 8 h, 24 h, and 48 h exhibited an inverse relationship between gelation time and fluid uptake. The highest fluid uptake was 743% at 4 h, while the lowest was 284% at 48 h. This trend was supported by the EDXRF results, which showed that the average concentration of copper with 4 h gelation was 67,566 mg/100 g and increasing gelation time led to a higher weight percent of copper ions of 48 h aerogel, 76,166 mg/100 g. As a result, the fluid uptake was reduced to 284% for samples gelated over 48 h.

This suggests that longer gelation times allowed for a higher concentration of copper ions to diffuse into the middle section of the aerogels, promoting a higher degree of crosslinking. The resulting denser microstructure, with finer micropores, was less capable of absorbing fluid or more resistant to fluid uptake.

#### 4.5.2. Evaluation of the Antibacterial Activity of Hydrogel and Aerogel Wound Dressing

**Figure 7:**
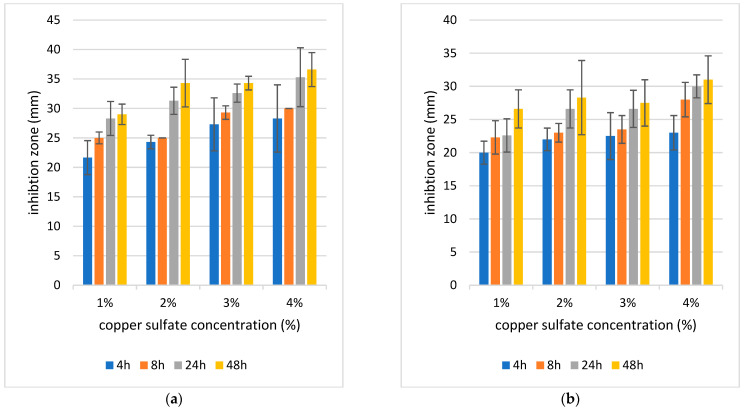
Inhibition zone of 4 concentrations of copper sulfate and 4 times of gelation hydrogel (4 h, 8 h, 24 h, 48 h) on (**a**) *S. aureus* (**b**) *E. coli.* The diameter of the sample is 18 mm, means ± SD, (*n* = 3).

**Figure 8:**
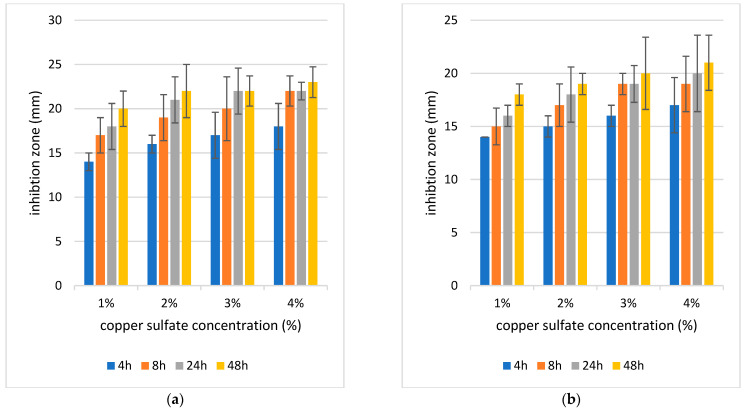
Inhibition zone of 4 concentrations of copper sulfate and 4 times of gelation aerogel (4 h, 8 h, 24 h, 48 h) on (**a**) *S. aureus* (**b**) *E. coli.* The diameter of the sample is 13 mm, means ± SD (*n* = 3).

As the inhibition zone indicates antibacterial activity, all samples of hydrogel and aerogel with different gelation times and copper concentrations showed remarkable antibacterial activity (Figure 7 and Figure 8).

Figure 7a shows how hydrogel response changes across different concentrations and time points. Both time and concentration had strong, independent effects on the results, while their interaction was not significant, meaning the trends with time were consistent across concentrations. Pairwise tests confirmed that several time intervals within the same concentration differed significantly, especially at stricter Bonferroni thresholds. Overall, the data suggest that increasing concentration and longer exposure both enhance the hydrogel response in a clear and systematic way. This analysis, as in Figure 7b, shows that both time and concentration significantly influenced the hydrogel response, with very strong statistical support (*p* ≈ 1.3 × 10^−5^ for time and *p* ≈ 0.0013 for concentration). The interaction between time and concentration was not significant (*p* ≈ 0.90), meaning the way the hydrogel changes over time is consistent across all concentrations. Pairwise comparisons across time within each concentration also revealed meaningful differences once corrected for multiple testing, highlighting that response values vary clearly as exposure time increases. In Figure 8a, both time (*p* ≈ 9.6 × 10^−6^) and concentration (*p* ≈ 0.0013) had strong and significant effects on the response. The interaction term was not significant (*p* ≈ 0.997), showing that the influence of time was consistent across concentrations. Pairwise comparisons between times within each concentration further confirmed that response values shift noticeably as exposure time increases, underscoring the strong role of temporal effects in the aerogel system. In Figure 8b, both time (*p* ≈ 4.7 × 10^−4^) and concentration (*p* ≈ 0.0014) significantly influenced bacterial response. The interaction between them was not significant (*p* ≈ 0.999), indicating that the effect of time was similar across all concentrations. Pairwise time comparisons within each concentration also revealed clear differences.

It was demonstrated that the antibacterial activity was stronger as the concentration of copper increased with *p*, which could be observed via the zone of inhibition (Figure 7 and Figure 8). Additionally, antibacterial activity was improved by prolonging the gelation period at all copper concentrations in hydrogel and aerogel. Therefore, samples of 4% and 48 h hydrogel and aerogel had the strongest antibacterial activity with mean inhibitory zones of 36 mm in hydrogel and 23 mm in aerogel for *S. aureus* and 31.6 mm in hydrogel and 21 mm in aerogel for *E. coli*, respectively. Since *S. aureus* and *E. coli* are the most frequent bacteria linked to wound infections, the ALG/HA aerogel and hydrogel with copper ions demonstrated an antibacterial effect against them. It was also observed that hydrogel samples show stronger antimicrobial activity than aerogel samples, which may be attributed to the higher water content of the former, as the presence of water in the hydrated network structure can lead to faster diffusion and release of copper ions. In contrast, aerogels’ dry structure may slow the release of copper ions due to stronger crosslinking and lower moisture content. Because copper ion release is responsible for antibacterial effects, variations in release kinetics—such as a faster and more readily available copper ion release from hydrogels compared to the aerogels—would explain the greater antibacterial activity observed for the hydrogel samples.

The samples exhibited higher antimicrobial activity against *S. aureus* than *E. coli* bacteria, which agreed with Klinkajon et al.’s findings. The cell wall of Gram-positive bacteria is thicker compared to that of Gram-negative bacteria, the small hydrated radius of Cu^2+^ promotes penetration into the bacteria’s intracellular matrix, and Gram-positive bacteria include teichoic acid, which is negatively charged, attracting positively charged substances (e.g., Cu^2+^). There are several ways in which Cu^2+^ can interact. It can destabilize the DNA double helix by binding to phosphate groups, form chelates with amino and carboxylate sites in proteins, and disrupt enzymatic activity [45]. Together, these mechanisms explain the strong antibacterial activity observed for Cu^2+^-crosslinked ALG/HA hydrogels and aerogels against both Gram-positive and Gram-negative bacteria such as including *S. aureus* and *E. coli*.

## 5. Conclusions

This study successfully developed copper-crosslinked ALG/HA aerogels as innovative wound dressing materials with enhanced antibacterial properties and fluid uptake capabilities. The physicochemical characterization of these aerogels revealed their unique structural and morphological features, which are influenced by copper ion concentration and crosslinking time. The antibacterial assays demonstrated significant inhibition against *S. aureus* and *E. coli*, highlighting the aerogels’ potential to combat infections effectively. Furthermore, the fluid uptake studies confirmed their ability to absorb wound exudates efficiently, making them suitable for maintaining a moist healing environment. The findings underscore the versatility of these bio-based aerogels in addressing critical challenges in wound management, including infection control and optimized healing conditions. This work paves the way for further exploration of biopolymer-based aerogels in advanced wound care applications and proves the concept of using bio-based aerogel and hydrogels as alternatives to conventional wound dressings. However, the current study lacks in vivo testing, which is necessary to fully assess the aerogels’ biocompatibility and wound healing efficacy under physiological conditions. Therefore, future work will focus on in vivo evaluations as well as optimizing the aerogel formulation for clinical applications.

## Figures and Tables

**Table 1:** The results of BET surface area of the prepared ALG-HA aerogel in this work, at different gelation times and copper concentrations; the results are illustrated as *n* = 3 ± SD.

2% ALG/HA (Gelation Time 4 h)		2% ALG/HA (Gelation Time 24 h)	
Cu wt.% in Gelation Bath	BET Surface Area m^2^/g	Cu% in Gelation Bath	BET Surface Area m^2^/g
1	325 ± 29	1	211 ± 34
2	294 ± 33	2	186 ± 26
3	263 ± 29	3	176 ± 30
4	246 ± 24	4	173 ± 16

## Data Availability

The raw data supporting the conclusions of this article will be made available by the authors on request.
